# Case Report: Hemophagocytic Lymphohistiocytosis and Non-Tuberculous Mycobacteriosis Caused by a Novel *GATA2* Variant

**DOI:** 10.3389/fimmu.2021.682934

**Published:** 2021-05-10

**Authors:** Thomas Mika, Deepak Vangala, Matthias Eckhardt, Paul La Rosée, Christoph Lange, Kai Lehmberg, Charlotte Wohlschläger, Saskia Biskup, Ilka Fuchs, Jasmin Mann, Stephan Ehl, Klaus Warnatz, Roland Schroers

**Affiliations:** ^1^ Department of Hematology and Oncology, Knappschaftskrankenhaus, Ruhr-University Bochum, Bochum, Germany; ^2^ Klinik für Innere Medizin II, Schwarzwald-Baar-Klinikum, Villingen-Schwenningen, Germany; ^3^ Medical Clinic, Research Center Borstel, Borstel, Germany; ^4^ Respiratory Medicine & International Health, University of Lübeck, Lübeck, Germany; ^5^ Clinic for Pediatric Hematology, University Medical Center Eppendorf, Hamburg, Germany; ^6^ Hematopathology Lübeck, Lübeck, Germany; ^7^ CeGaT and Practice for Human Genetics, Tübingen, Germany; ^8^ Institute for Immunodeficiency, Center for Chronic Immunodeficiency, Medical Center, Faculty of Medicine, University of Freiburg, Freiburg i.Br., Germany; ^9^ Department of Rheumatology and Clinical Immunology, Medical Center, Faculty of Medicine, University of Freiburg, Freiburg i.Br., Germany; ^10^ Center for Chronic Immunodeficiency, Medical Center, Faculty of Medicine, University of Freiburg, Freiburg i.Br., Germany

**Keywords:** hemophagocytic lymphohistiocytosis, non-tuberculous mycobacteriosis, GATA2, CD107a, allogeneic hematopoietic stem cell transplantation

## Abstract

Hemophagocytic lymphohistiocytosis (HLH) is a disorder of uncontrolled immune activation with distinct clinical features including fever, cytopenia, splenomegaly, and sepsis-like symptoms. In a young adolescent patient a novel germline *GATA2* variant (NM_032638.5 (GATA2): c.177C>G, p.Tyr59Ter) was discovered and had resulted in non-tuberculous mycobacterial (NTM) infection and aggressive HLH. Strikingly, impaired degranulation of cytotoxic T-lymphocytes (CTL) and natural killer (NK)-cells was detected in CD107a-analyses. The affected patient was treated with HLA-matched unrelated alloHSCT, and subsequently all hematologic and infectious abnormalities including HLH and NTM resolved. This case supports early alloHSCT in GATA2 deficiencies as curative approach regardless of active NTM infection. Future studies on GATA2 c.177C>G, p.Tyr59*Ter might unravel its potential role in cytotoxic effector cell function and its contribution to HLH pathogenesis.

## Introduction

Hemophagocytic lymphohistiocytosis (HLH) is a devastating disorder of uncontrolled immune activation with features resembling systemic inflammatory response syndrome (SIRS). Originally observed in pediatric patients, HLH has been increasingly recognized also in adults. HLH results from aberrantly activated macrophages and cytotoxic T-lymphocytes (CTL). The clinical hallmarks are fever, cytopenia, splenomegaly, and SIRS-like features often including liver-dysfunction. In contrast to children, HLH in adults presents predominantly as acquired (secondary) syndrome triggered by infections, malignancies, or autoimmune disorders (1).

Genetic (primary) HLH is considered to be rare in adults, however, variants in distinctive HLH-associated genes previously have been reported in up to 14% of affected patients (2). Lymphocyte cytotoxicity and natural-killer (NK) cell function are impaired in primary HLH. Accordingly, immunophenotypic and functional analysis of these effector cells together with guided genetic testing are recommended in selected adult patients (1).

GATA binding protein 2 (GATA 2) is a transcription factor and key component in hematopoiesis and stem cell biology. *GATA2* variants cause heterogeneous abnormalities including hematologic, immunologic, and dermatologic diseases. In GATA2 deficiency, B-lymphocytopenia, NK-lymphocytopenia, and monocytopenia are striking findings, often accompanied by opportunistic infections such as non-tuberculous mycobacteriosis (NTM). Immunodeficiency as caused by *GATA2* variants can be the basis of HLH, as has been described in single cases of severe primary Epstein-Barr virus (EBV) and cytomegalovirus (CMV) infections (3, 4).

Here, we describe the medical history and molecular work-up of a young woman with persistent fever, splenomegaly, lymphadenopathy, and pancytopenia. The recognition of an HLH-like disorder was followed by the diagnosis of systemic NTM infection with *Mycobacterium avium*. Finding a markedly impaired degranulation of CTL and NK-cells as detected in CD107a-analyses, advanced genetic testing was performed and finally revealed a germline heterozygous *GATA2* variant (c.177C>G, p.Tyr59*Ter). The resulting GATA2 deficiency was deemed predisposing to poor NTM infection control resulting in persistent immune stimulation with subsequent HLH. This ultimately supported the choice of allogeneic hematopoietic stem cell transplantation (alloHSCT) as successful curative treatment approach in the reported patient.

## Case Presentation - Tests and Therapy

**Figure 1:**
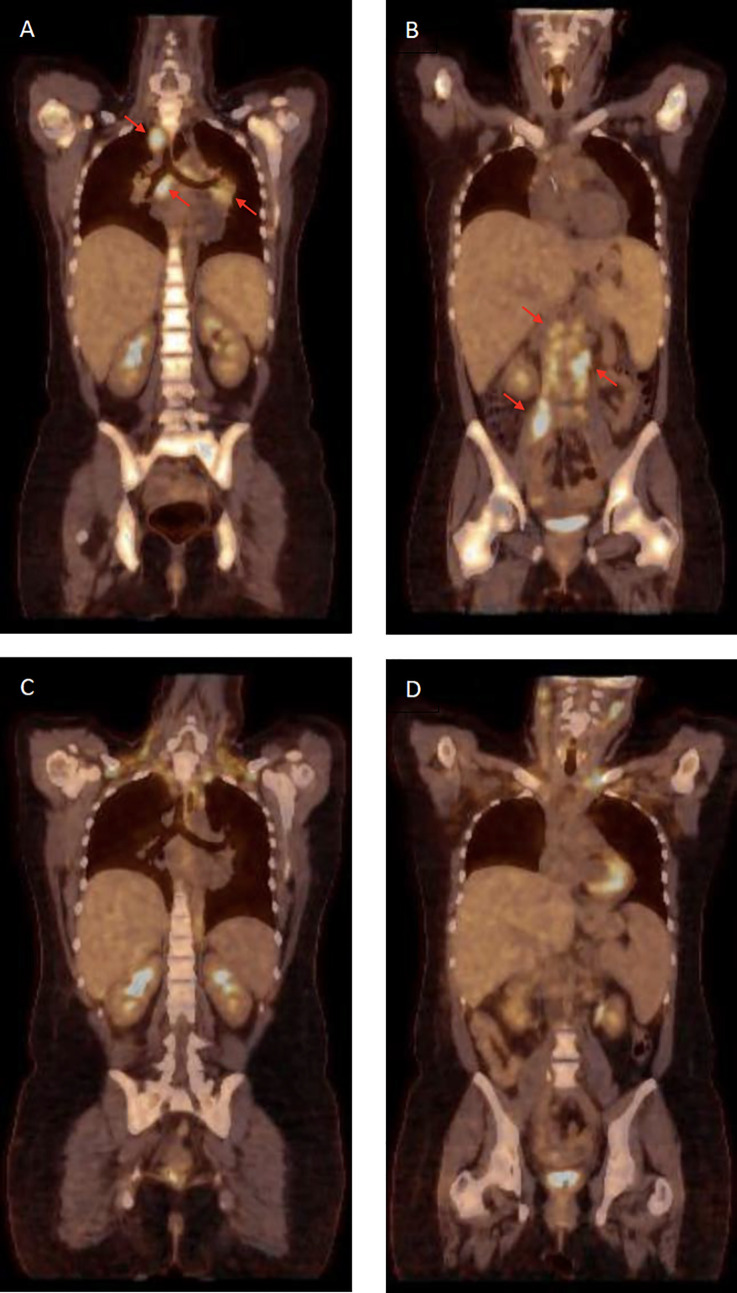
Positron emission tomography–computed tomography (PET-CT). **(A, B)** Disseminated lymphadenopathy due to non-tuberculous mycobacterial (NTM) infection (red arrows) and bone-marrow activation and hepatosplenomegaly due to hemophagocytic lymphohistiocytosis (HLH) at diagnosis. **(C, D)** Control PET-CT following allogeneic hematopoietic transplantation (day +240) and after 12 months of tuberculostatic therapy demonstrating complete remission of NTM-related lymphadenopathy and remission of HLH-associated hematopoietic activation.

In brief, the 29-year old student presented to her general practitioner with recurrent fever up to 40°C in December 2019. Medically, she and her relatives had an uneventful past. After fever persistence for more than two weeks she was admitted to hospital for an extensive work-up. In clinical and ultrasound examinations, abdominal lymph node enlargements and a marked hepatosplenomegaly were noticed. These findings were confirmed in positron emission computer tomography (PET-CT) scanning (**Figures 1A, B**). Her blood counts showed pancytopenia (WBC 1.5 x 10^9^/l, neutrophils 1.0 x 10^9^/l, lymphocytes 0.35 x 10^9^/l, monocytes 0.01 x 10^9^/l, hemoglobin 76 g/l, platelets 78 x 10^9^/l), and additional lab testing revealed hyperferritinemia (3860 g/l), hypertriglyceridemia (4.56 mmol/l), increased serum levels of aspartate aminotransferase (AST 79 U/l), soluble interleukin-2 receptor (sIL-2R, 2710 U/l), and lactate dehydrogenase (LDH 626 U/L). Although fibrinogen was normal (5.1 g/l), a diagnosis of HLH was made based on HLH-2004 criteria [7/8 fulfilled including impaired NK-cell function, see below; (5)] and an HScore of 239 points [98-99% probability of hemophagocytosis; (6)], respectively.

After ruling out Leishmania spp., HIV, EBV, and CMV infections by serology and PCR (peripheral blood and bone marrow, BM), biopsies of retroperitoneal lymph nodes were taken. Surprisingly, histopathology did not show lymphoma but demonstrated granulomatous lymphadenitis including acid-fast bacteria, which were further classified as *Mycobacterium avium* by genotyping (MycoDirect 1.7 LCD array, Chipron, Germany). *M. avium* was also detected in BM and cultured from bronchoalveolar lavage samples, giving the diagnosis of NTM infection triggering HLH. In BM cytology and histopathology no signs of myelodysplasia were identified.

**Figure 2:**
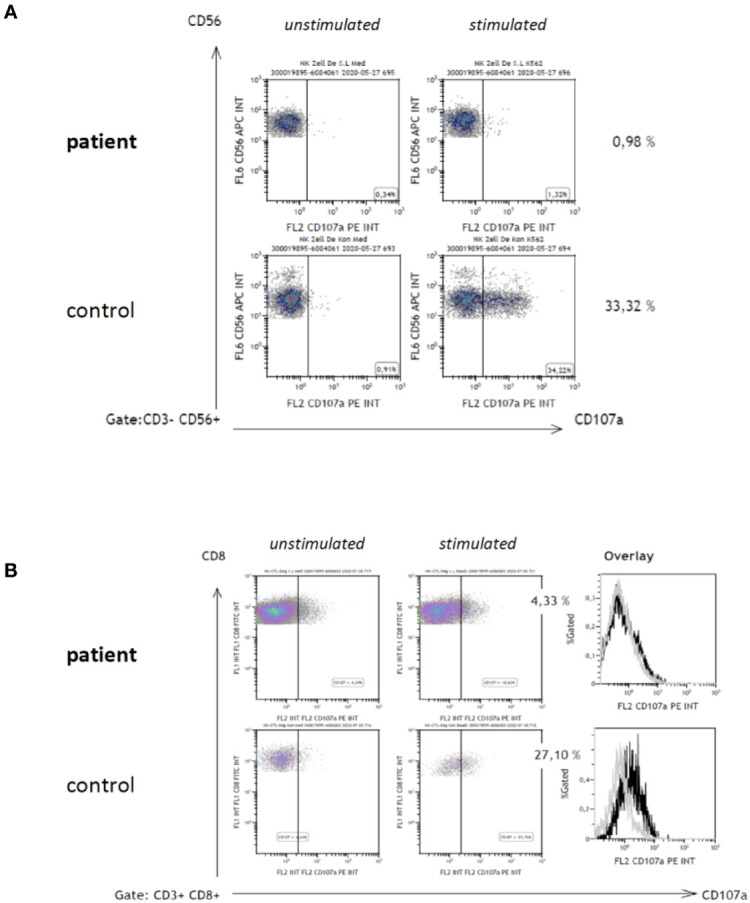
CD107a degranulation assay. Degranulation of CD56+ NK-cells **(A)** and CD8+ CTL **(B)** was examined as previously published^7^. In brief, CD107a-expression on cell surfaces was analyzed by flow-cytometry in resting cells (**A**, **B;** *left dot plot diagrams*) and subsequent to 48 h stimulation with interleukin 2 (IL-2) of NK-cells (**A**, *right dot plot diagrams*), and phytohemagglutinin (PHA)/IL-2 of CD8+ T-cells (**B**, *right dot plot diagrams*), respectively.

Treatment with amikacin, azithromycin, ethambutol and rifampicin was initiated promptly, however, the fever and other HLH-signs persisted. Only after intensive immunosuppressive therapy including high-dose dexamethasone, ciclosporin A, intravenous immunoglobulins, and repetitive courses of etoposide (75 mg/m^2^, twice weekly) the HLH-related symptoms could temporarily be ameliorated. The cause for HLH and NTM was further investigated including CTL and NK-cell function analysis in CD107a-assays (7). These indicated significant degranulation impairments in both effector cell types (**Figure 2**). Perforin expression was normal, and genetic variants in *UNC13D*, *STXBP2*, *RAB27A*, and *STX11* were excluded in Sanger sequencing. Subsequently, next generation sequencing (NovaSeq 6000 NGS platform) of genes associated with NTM infections [*CYBB, GATA2, IFNGR1/R2, IL12B, IL12RB1/RB2, IL23R, IRF8, ISG15, JAK1, RORC, SPPL2A, STAT1, TYK2*; www.CEGAT.de; (8)] was performed. As result, a novel *GATA2* variant (c.177C>G, p.Tyr59Ter) in heterozygous form was identified (DNA from peripheral blood and mucosa cells).

Considering disseminated NTM infection and secondary HLH resulting from primary immunodeficiency due to germline GATA2 deficiency, we aimed for alloHSCT as curative therapy. An HLA-matched unrelated donor (MUD 10/10) was identified, and the patient underwent alloHSCT following reduced-intensity conditioning with fludarabine (150 mg/m²) and treosulfan (30 g/m²) in September 2020. Graft-versus-Host disease (GvHD) prophylaxis consisted of post-transplant cyclophosphamide (100 mg/kg), mycophenolate-mofetil, and ciclosporin. Prior to alloHSCT, the antimycobacterial treatment was switched to azithromycin, clofazimine, and ethambutol. The fever and HLH-signs completely resolved after full hematopoietic engraftment. In the absence of GvHD, ciclosporin was ceased at day +98 post-alloHSCT. Following diagnosis of moderate hepatotoxicity, which appeared drug-related in a liver biopsy, all antibiotics were stopped 6 months after alloHSCT and 1 year after disease onset, respectively. PET-CT scanning at that time demonstrated complete remission of all inflammatory manifestations (**Figures 1C, D**).

## Discussion

HLH is a hyperferritinemic hyperinflammatory syndrome with a common immunologic pathway and inability of the immune system to adequately restrict stimulatory effects towards various triggers (1). In adults, infections and malignancies, primarily lymphomas, are the most frequent initiators of HLH, as has recently been confirmed by our group in a large collaborative analysis (9). Our young adult patient presented with new-onset aggressive HLH requiring straightforward investigation of underlying disorders. Considering her mediterranean origin leishmaniasis was ruled out in addition to infections with HIV and herpesviridae. PET-CT scanning was highly suggestive of malignant lymphoma, but histopathology including molecular diagnostics and culture established the final diagnosis of *Mycobacterium avium* infection.

While secondary HLH has been repeatedly described in relation to typical tuberculosis, occurrence of HLH and NTM such as *M. avium* remains extremely rare (10). Noteworthy, there is no report on both conditions in the context of primary GATA2 deficiency until today. More often, gene sequence variants associated with adult HLH occur in *PRF1*, *STXBP2*, *SH2D1A*, and *UNC13D*, respectively (2).

In our patient, low CD107a-expression on stimulated CTL and NK-cells (**Figure 2**) was indicative of impaired cytotoxic degranulation. As previously published, quantification of CD107a on surfaces of cytotoxic lymphocytes in standardized robust protocols has a high sensitivity and specificity for the diagnosis of HLH in conjunction with genetic disorders of granule exocytosis (7, 11). However, none of the classic genes associated with exocytosis and primary HLH were mutated in the reported patient.

Disseminated NTM in adolescents and young adults can be caused by acquired autoantibodies against interferon-γ and by primary immune defects due to variants of *IL-12RB1, IFNGR1/R2*, *STAT1LOF*, and *GATA2*, respectively (8). Compatible with MonoMAC-syndrome due to GATA2 deficiency, our patient not only suffered from mycobacterial infection but also presented with monocytopenia, B-lymphopenia (9/μl), and NK-lymphopenia (50/μl) (3, 12, 13).

Interestingly, reduced NK-cell cytotoxicity and specific loss of the CD56^bright^ NK-subset have previously been demonstrated in GATA2 deficiency, but this was linked to impaired differentiation of cytotoxically active NK cells rather than failure of degranulation (14). Our patient repeatedly showed absent CD107a-expression in fresh NK-cells, possibly related to the newly identified *GATA2* variant c.177C>G, p.Tyr59*Ter. It is plausible to assume that the severe functional impairment contributed to the NTM infection and the associated HLH. As the family history and *GATA2* genetic analysis of first-degree relatives revealed no further affected individuals, the variant of *GATA2* was *de novo*. This genetic variant leads to a premature stop codon and most probably results in nonsense-mediated mRNA decay. Certainly, further unraveling the molecular mechanisms and role of GATA2 in cytotoxic granule exocytosis will be of future interest.

Patients with primary immunodeficiency due to *GATA2* variants and NTM infections have poor responses to antimicrobial drugs alone (8, 12). In line with this, the lymphadenitis and HLH were both refractory against combined antimycobacterial and immunosuppressive therapy in our patient. Cure in these situations necessitates alloHSCT as definite treatment (8, 15). AlloHSCT in GATA2 deficiency including MonoMAC-syndrome has been reported in case series and within a prospective clinical trial, describing a disease-free survival rate of 86% following a busulfan/fludarabine-based conditioning regimen (15). In our patient, alloHSCT was challenging because of florid disseminated NTM infection and HLH requiring intensive immunosuppressive therapy. However, short duration of clinical disease and absence of myelodysplasia, myeloid leukemia, and additional infections such as human papilloma virus were favorable. Hence, we chose a reduced-intensity conditioning regimen based on treosulfan/fludarabine and post-transplant cyclophosphamide to facilitate reliable hematopoietic engraftment with acceptable risks of infectious complications and GvHD, respectively.

In summary, we describe a germline *GATA2* variant as the basis of disseminated NTM infection and HLH. The affected patient was successfully treated with HLA-matched unrelated alloHSCT in parallel to prolonged antimicrobial treatment. Subsequent to alloHSCT all hematologic abnormalities and signs of NTM infection and HLH resolved, and the patient experienced full clinical recovery. This case supports early alloHSCT in GATA2 deficiencies as curative approach regardless of active systemic NTM infection. Further studies on the novel *GATA2* variant (NM_032638.5 (GATA2): c.177C>G, p.Tyr59Ter, pathogenic) might help understanding the potential role of GATA2 in degranulation of cytotoxic effector cells and HLH pathogenesis.

## Accession Gene Database

ClinVar accession for the novel GATA2 variant is: https://www.ncbi.nlm.nih.gov/clinvar/variation/1013219/ Variation ID: 1013219 Accession: VCV001013219.1.

## Data Availability Statement

The original contributions presented in the study are publicly available. This data can be found here: https://www.ncbi.nlm.nih.gov/clinvar/variation/1013219/.

## Ethics Statement

The patient agreed on publication of this study and gave her written informed consent.

## Author Contributions

TM and RS collected all data and wrote the manuscript. TM, DV, ME, PR, KL, CL, JM, SE, KW, and RS guided and discussed all clinical decisions. CW, SB, and IF performed all diagnostic tests including histopathology, flow-cytometry, and genetic analyses. All authors contributed to the article and approved the submitted version.

## Conflict of Interest

The authors declare that the research was conducted in the absence of any commercial or financial relationships that could be construed as a potential conflict of interest.
